# Regulatory Implications for considering flexible resources in network expansion planning: main lessons from regional cases in the H2020 FlexPlan project

**DOI:** 10.12688/openreseurope.15873.1

**Published:** 2023-09-15

**Authors:** Andrei Morch, Gianluigi Migliavacca, Aleksandr Egorov, Hakan Ergun, Dario Siface, Giorgia Lattanzio, Iver Bakken Sperstad

**Affiliations:** 1 Energy Systems, SINTEF Energi AS, Trondheim, Trøndelag, NO-7034, Norway; 2 Energy Systems Development, Ricerca sul Sistema Energetico – RSE S.p.A., Milano, IT-20134, Italy; 3 Centro de Investigação em Energia REN - State Grid, S.A., Sacavém, PT-2685-038, Portugal; 4 Department of Electrical Engineering, Katholieke Universiteit Leuven, Leuven, Flanders, BE-3000, Belgium

**Keywords:** Grid Expansion Planning Tool, Flexible Resources, Demand Response and Energy Storage

## Abstract

The FlexPlan Horizon2020 project aimed at establishing a new grid planning methodology which considers the opportunity to introduce new storage and flexibility resources in electricity transmission and distribution grids as an alternative to building new grid elements, in accordance with the intentions of the European Commission regulatory package "Clean Energy for all Europeans". FlexPlan creates a new innovative grid planning tool which is intended to go beyond the state of the art of planning methodologies by including the following innovative features: assessment of best planning strategy by analysing in one shot a high number of candidate expansion options provided by a pre-processor tool, simultaneous mid- and long-term planning assessment over three grid years (2030-2040-2050), incorporation of full range of cost benefit analysis criteria into the target function, integrated transmission distribution planning, embedded environmental analysis (air quality, carbon footprint, landscape constraints), probabilistic contingency methodologies in replacement of the traditional N-1 criterion, application of numerical decomposition techniques to reduce calculation efforts and analysis of variability of yearly RES and load time series through a Monte Carlo process. Six regional cases covering nearly the whole European continent are developed in order to cast a view on grid planning in Europe till 2050. The final step in FlexPlan was formulating guidelines for regulators and planning offices of system operators, by indicating to what extent system flexibility can contribute to reduce overall system costs (operational and investment) yet maintaining current system security levels and which regulatory provisions could foster such process. This paper focuses on the regulatory issues, which were uncovered during the initiation phase and of the project and refined in throughout six regional cases. In order to substantiate this, the paper explains in brief the developed and applied FlexPlan methodology and its testing in six regional cases.

## Plain language summary

The FlexPlan Horizon 2020 project developed a novel methodology for transmission and distribution grid expansion planning, which considers use flexible resources e.g., energy storage and demand response, as an alternative to the conventional approach i.e., building new grid elements. The methodology was implemented in a dedicated software tool and extensively tested in six regional cases, covering a major part of Europe. Throughout the whole project much attention was paid to the existing and forthcoming regulatory aspects to ensure that the developed methodology complies with them. Furthermore, based on the outcomes and lessons learned throughout the regional cases, the study has suggested several proposals i.e., regulatory guidelines for improvement of the existing regulatory framework in order to remove the barriers and pave the road for more active use of flexible resources in the grid expansion planning, and thereby to achieve more efficient operation of the distribution and transmission networks with high share of renewable generation.

## Introduction

The Renewable Energy Directive (REDII) ^1^ introduced a very ambitious binding target of 32% on the share of energy from renewable energy sources (RES) for the year 2030. Furthermore, with the 2030 Climate Target Plan in 2020 ^2^, the European Commission (EC) proposes to raise the EU's ambitions for reducing greenhouse gas emissions to at least 55% below 1990 levels by 2030 ^3^. In July 2021 the commission presented a revised version of REDII aiming to increase the current target to at least 40 % of renewable energy sources by 2030. The most recent REPower EU plan ^4^ addressed the unexpected political and economic turmoil caused by the outbreak of the war in Ukraine, which resulted in unprecedented energy crises with extremely high prices for electricity in Europe and has proposed to further increase the share of renewables to 45%. 

Such massive RES deployment will make future transmission and distribution (T&D) grid planning more complex and affected by uncertainty. Grid investments are capital intensive, and the lifetime of transmission infrastructure spans over several decades: due to rapidly changing scenario hypotheses, when a new line is commissioned, the foreseen benefits could no longer justify the corresponding investment.

For this reason, it becomes increasingly important to optimize planning of grid infrastructure while finding other ways to compensate for peak flows in the grid. On this pathway, storage can provide a good alternative to building new lines, when placement of storage devices in strategic grid locations could prevent temporary line overloading, and thus serve as a good alternative to building new lines. A similar role could be also taken by flexible consumption (e.g., deferrable consumption), especially when considering big industrial loads and tertiary infrastructures. The opportunity to consider the usage of flexible resources as support for grid planning has been clearly highlighted in the European Directives (e.g., Internal Electricity Market (IEM) Directive ^5^ of the package “Clean Energy for All Europeans”). However, a survey conducted in 2020 ^6^ showed that there was no common practice for this, neither from a methodologic nor technical viewpoint.

All these aspects are covered by the FlexPlan Horizon 2020 project ^7^, which has developed and tested a new grid planning methodology and dedicated software tool considering the opportunity to introduce new storage and flexibility resources in electricity T&D grids as an alternative to building new grid elements. The software tool has been extensively tested in six regional cases (RCs) covering nearly the whole European continent (Iberian Peninsula, France and Benelux, Germany Switzerland and Austria, Italy, Balkan Countries and Nordic Countries), aimed at demonstrating the application of the tool on real scenarios as well as at casting a view on grid planning in Europe till 2050.

The present paper focuses on outcomes of from one of the activities in FlexPlan, namely “Regulatory analysis”, which was conducted in three main steps:

•  The initial screening of the Pan-European regulatory framework aimed to uncover the open issues and shortcomings, plus to ensure that the project outcomes comply with the overall Pan-European political targets

•  Evaluation of the six RCs accomplished in FlexPlan from the regulatory point of view and, based on this, definition of the main regulatory limitations and barriers

•  Development of final recommendations and guidelines for the Pan-European level regulation 

In order to justify the conclusions, the study also presents the main highlights from both FlexPlan methodology and the accomplished RCs from two other activities in the project.

## Methods

### The initial FlexPlan screening study

A comprehensive assessment of the Pan-European regulatory framework was carried out at the beginning of the project. The intention was to ensure that the project outcomes comply with the overall Pan-European political targets and thereby to set an optimal environment for the real implementation of the planning tool realized by the FlexPlan project.

The first step applied qualitative evaluation methods, based on data collected through literature screening and survey-based research. The activity followed a stepwise approach, which is presented in the FlexPlan report D6.1 ^8^, where the activity was divided into two parallel streams: one carried out a screening of a set of documents selected by the project group, while another complemented the former by a reference to the existing practices at both transmission and distribution system operators (TSOs and DSOs) based on a survey. The survey involved three European TSOs and four DSOs.

The screening covered a selection of the relevant documents, issued by several types of stakeholders, including the EC, the European Network of Transmission System Operators for Electricity (ENTSO-E) and the interest organisations representing DSOs. The study focused on a pre-defined selection of issues, which have critical importance for FlexPlan project and are called "topics of interest". These topics represent either some key assumptions that will have to be made within the project activities, or/and some attributes, which can be directly or indirectly decisive for the development and later for the implementation of the project outcomes. 

A selection of the main conclusions from analysis of the regulatory status quo and strategies in Europe are presented below. For complete results, see the FlexPlan report D6.1 ^8^.

***Requirements related to consideration of flexible resources in planning.*** This section summarises the results obtained from the screening process above. The importance of the flexible resources was clearly stated in the IEM Directive ^5^ The document includes a specific section (Art.32) dedicated to incentives for use of flexibility sources in distribution, stating that the distribution network development plan shall also consider demand response, energy efficiency, energy storage facilities or other resources that the DSO has to use as an alternative to system expansion. Furthermore, the same document defines that, when elaborating the Ten-Year Network Development Plan (TYNDP), TSOs shall fully consider the potential for the use of demand response, energy storage facilities or other resources as alternatives to system expansion. The EC Regulation 2019/943 ^9^ on the Internal Market for Electricity, which is linked to the above-mentioned directive, states that to integrate the growing share of renewable energy, the future electricity system should make use of all available sources of flexibility, particularly demand side solutions and energy storage. In ENTSO-E's 3 ^rd^ guideline for cost benefit analysis (CBA) of grid development projects ^10^, flexibility of demand is considered as a consistent part of the estimation of socio-economic welfare.

The project concluded that there was a clear indication from the present regulatory framework supported by a broad agreement across different stakeholders, that flexible resources should be used as a viable resource for the operation of the power system and thus it should be considered in the planning procedures of the power grid. 

***Ownership and operation of energy storage.*** The study specifically highlighted the importance of this issue with regards to the establishment of a regulation to support a future planning methodology taking into consideration the role of storage and flexibility in the FlexPlan methodology. The most recent version of IEM Directive ^5^ indicates the official position of the EC regarding ownership of energy storage facilities by DSOs and TSOs, respectively. The document reaffirmed the position stated in the previous drafts of the directive, which, as a general rule, does not allow system operators (both TSOs and DSOs) to own, develop, manage, or operate energy storage facilities. However, Art. 36 and Art. 54 of the same document state that DSOs and TSOs are allowed to own, operate, or manage such devices, among other conditions, if these devices are “are fully integrated network components and the regulatory authority has granted its approval”.

***Rules for allocation of costs and incomes between TSOs and DSOs in new common investment projects.*** From the transmission side, ENTSO-E developed a guideline for CBAs of grid development projects ^10^, ensuring a common framework for multi-criteria CBA for TYNDP projects. However, there are no commonly agreed rules for allocation of costs between TSOs and DSOs in common investment projects.

The survey results ^10^ indicated that the present practice was based on a split of costs at transmission system level. However, this practice may be reconsidered in case flexibility resources from distribution networks will be actively employed and coordinated for the provision of system services to TSOs. For that time there was no regulatory framework applicable to this case.

***Costs functions representing reliability in Cost and Benefit Analysis.*** The study indicated that the main challenge is to represent reliability in monetary terms. The commonly used key indicator for reliability is the lost load, which is monetised via the value of lost load indicator (VOLL). According to ENTSO-E's CBA guideline the value for VOLL that is used during project assessment should reflect the real cost of outages for system users, hence providing an accurate basis for investment decisions. It is also stated that the experience has demonstrated that estimated values for VOLL vary significantly in dependency of geographic factors, differences in the nature of load composition, the type of affected consumers, and the level of dependency on electricity in the impacted geographical area, differences in reliability standards, the time of year and the duration of the outage.

***Priorities for sharing of resources between TSO and DSO.*** The IEM Directive ^5^ defines that DSOs shall cooperate with TSOs for the effective participation of market participants connected to their grid in retail, wholesale, and balancing markets. Delivery of balancing services stemming from resources located in the distribution system shall be agreed with the relevant TSO.

However, further screening and the survey of the present practice carried out by FlexPlan indicated that at present there is no common regulatory or practice background allowing to draw clear conclusions on this topic. The necessity of defining this is clearly highlighted both at the institutional level and by the stakeholders.

***Conclusions from the screening.*** The first step concluded that there were strong regulatory signals prompting European system operators to consider flexible resources as a new important active subject in the grid expansion planning process. This once again strengthened the importance and proper timing of the FlexPlan project, both for testing new innovative grid planning methodologies coping with the present challenges, for the comprehensive scenario assessment up to 2050 and for the final synthesis of the results into regulatory guidelines brought to the attention of National Regulators and the Commission.

### The FlexPlan methodology

The aim of the FlexPlan methodology is to perform network planning form a holistic perspective allowing to find trade-offs between classical and flexible grid expansion options while also considering their environmental impact. The planning methodology is applied both to transmission and distribution networks, and as such, distribution grid flexibility investments are used as a means to alleviate possible congestion in the transmission network. The core of the methodology is a multi-period, stochastic mixed- integer linear problem which determines the optimal network expansion options under a multitude of future scenarios and operational conditions ^11^. 

**Figure 1.**
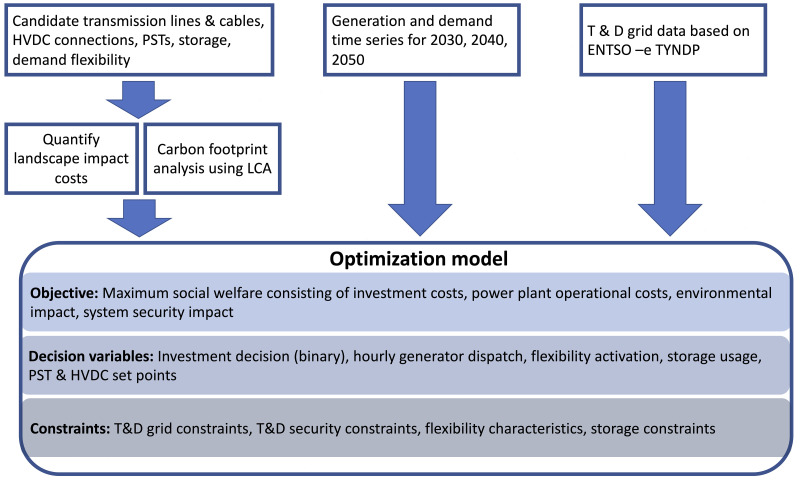
Overview of the FlexPlan optimal planning model.

Figure 1 shows the structure of the optimisation model and the used input data. A set of possible grid expansion options, e.g., alternating current (AC) and direct current (DC) transmission assets, AC distribution assets, demand flexibility and storage investments are provided as an input for the tool. These expansion candidates are characterised both technically and economically by the FlexPlan pre- processor. Further, the installed power plant capacity, time series for nodal generation and demand, grid topology and impedance data are used as input. An hourly resolution of the problem is considered over three investment years of the planning horizon, namely 2030, 2040 and 2050.

The objective of the developed optimisation model is to maximise social welfare, which is formulated as a cost minimisation problem. The considered costs are the investment costs for transmission and distribution grid expansion, the operational costs bound to generation dispatch and demand flexibility, as well as environmental impact costs consisting of CO _2_ emission, air quality impact and landscape impact costs. The details of the objective function can be found in the FlexPlan report D1.2 ^11^ and in Migliavacca *et al.* ^12^. The decision variables of the problem are, the investment decisions for the expansion options, hourly dispatch of generation, storage, and demand flexibility. The optimisation problem is constrained by linearised power flow equations, and the operational limits of the technical equipment.

The resulting optimisation problem contains millions of decision variables and constraints, and as such, to obtain a tractable implementation, several model simplifications have been applied which are listed below:

- Time series clustering for reducing the size of the problem, presented in FlexPlan report D1.1 ^13^
- Sequential solution of the problem over the planning horizon instead of a one-shot optimisation.
- Decomposition of transmission and distribution grids, using surrogate models for the distribution grid, presented in M. Rossi et al paper at CIRED2021 ^14^
- Using a representative set of distribution grids per analysed RC.

A proof-of-concept of the FlexPlan planning methodology has been implemented as an open-source software package – FlexPlan ^15, 16^.

### Testing the FlexPlan methodology in the RCs

This section presents a summary of the methodology used to obtain the results for the six RC studies:

- Iberian RC (covering the Iberian Peninsula)
- France and Benelux RC (covering Belgium, the Netherlands and Luxemburg)
- Switzerland, Austria, and Germany RC
- Italy RC
- Balkan Region RC
- Northern RC (covering Norway, Finland, Sweden, and Denmark)

The overall approach included four main steps:

- Obtaining the initial input data, which includes the collection of relevant information such as grid data, scenario time series for load and generation to be simulated and environmental impact data
- Solving the optimal power flow (OPF) in the non-expanded grid models and identifying the existing congestions, Lagrange multipliers (LMs) related to the congestions, operational costs including load and generation curtailment costs and other relevant results
- Based on LMs, which are ranked by the severity of congestions, the pre-processor proposes the set of possible candidates
- Solving the grid expansion planning problem by the FlexPlan planning tool, which uses both the results from non-expanded OPF and the set of possible candidates to identify the best investment decisions in order to minimize the total costs of the RC

**Figure 2.**
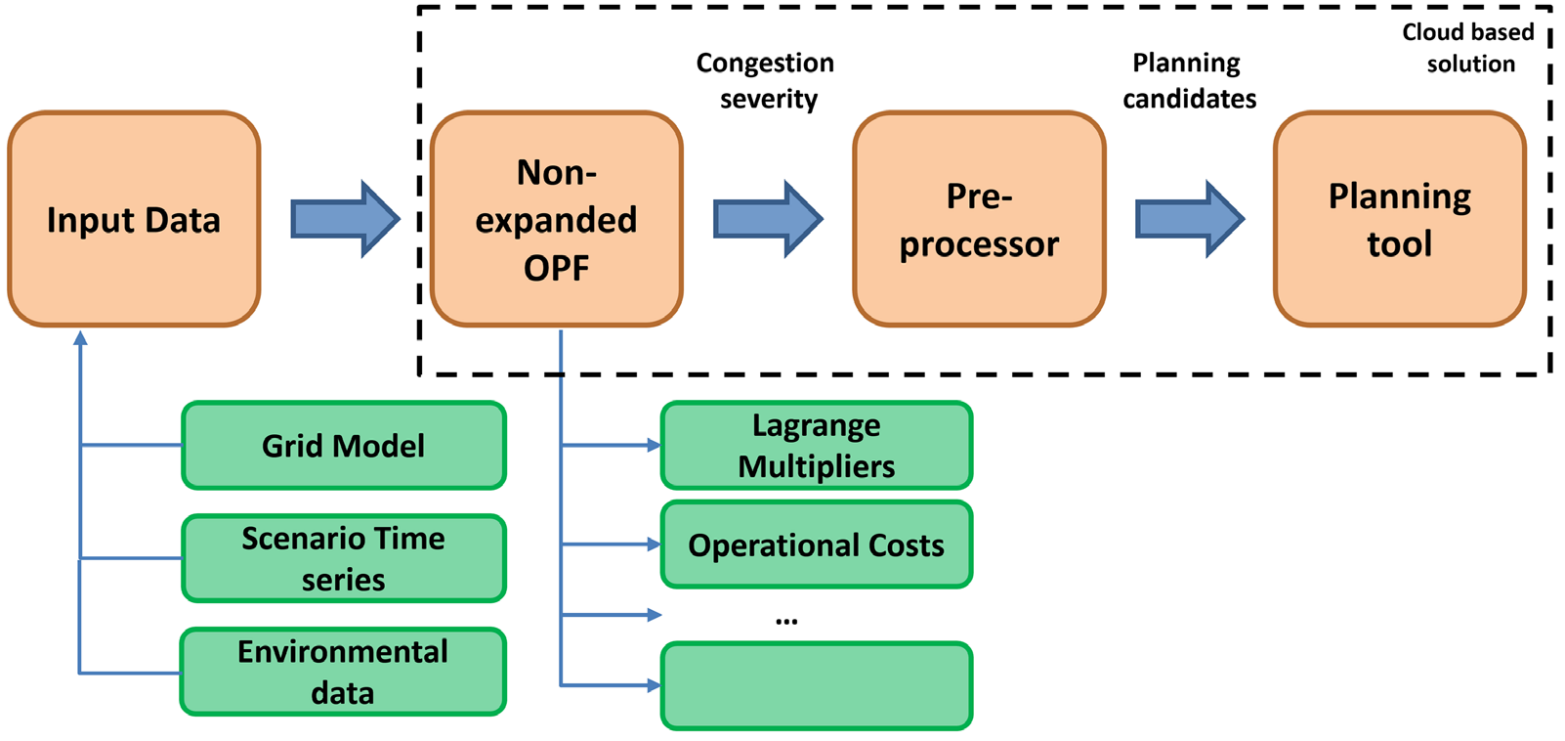
The FlexPlan methodology.

The overview of the methodology is shown in Figure 2.

The grid models consist of the transmission network data that was obtained mainly from ENTSO-E ^17^ by signing a non-disclosure-agreement and complemented with the data from open-source models like the PyPSA-Eur model ^18^, local TSOs data and regulators’ data to fill the gaps. It also includes the distribution network data, which is based on the aggregated power profiles of distributed load and generation, and their spread over the synthetic distribution networks to model the connected loads and generation units at each node.

The scenario time series data is defined at the target years 2030, 2040 and 2050, as it is the time horizon to achieve the reduction of emission to net-zero. The main source for the scenarios is taken from the TYNDP 2020 ^19^ and complemented with the TYNDP 2018 ^20^ and Mid-Term Adequacy Forecast (MAF) 2018 ^21^ to fill the gaps in the data. These scenarios were also validated with the “A Clean Planet for All” strategy from the European Commission ^22^ as a comparative source.

Additionally, environmental data is necessary to evaluate the environmental impact of the grid extension across the transmission and distribution networks. It consists of three main aspects:

•  Carbon footprint impact, which is calculated for all possible candidates used in the FlexPlan planning tool

•  Landscape impact, which considers the cost of installation and visual impact for overhead and cable transmission systems for a variety of geographical areas

•  Air quality impact, which determines the health impact of emissions from conventional generation based on historical data sets for different geographical regions and climatic conditions

In order to solve the grid expansion planning problem, the FlexPlan tool implements a grid expansion optimization engine capable of assessing flexibility source candidates along with conventional grid reinforcement candidates, such as new lines, cables, and transformers. Flexibility sources are meant to include different storage technologies as well as demand-side management. In addition, the tool assesses candidates in both transmission and distribution networks, which provides the possibility of optimizing the procurement of flexibility on both sides of the network simultaneously.

The list of candidates is provided by the FlexPlan pre-processor, which is an external application based on a heuristic algorithm which can propose investment candidates for a network expansion problem based on the outputs of an optimal power flow from a given network. The pre-processor tool uses the locational marginal pricing (LMP) information from optimal power flow calculations which are carried out on the non-expanded network over the course of a full year. Based on this information and the network characteristics, the most impactful grid expansion options with respect to reducing LMPs are proposed, considering the technical characteristics and costs of the expansion options.

**Figure 3.**
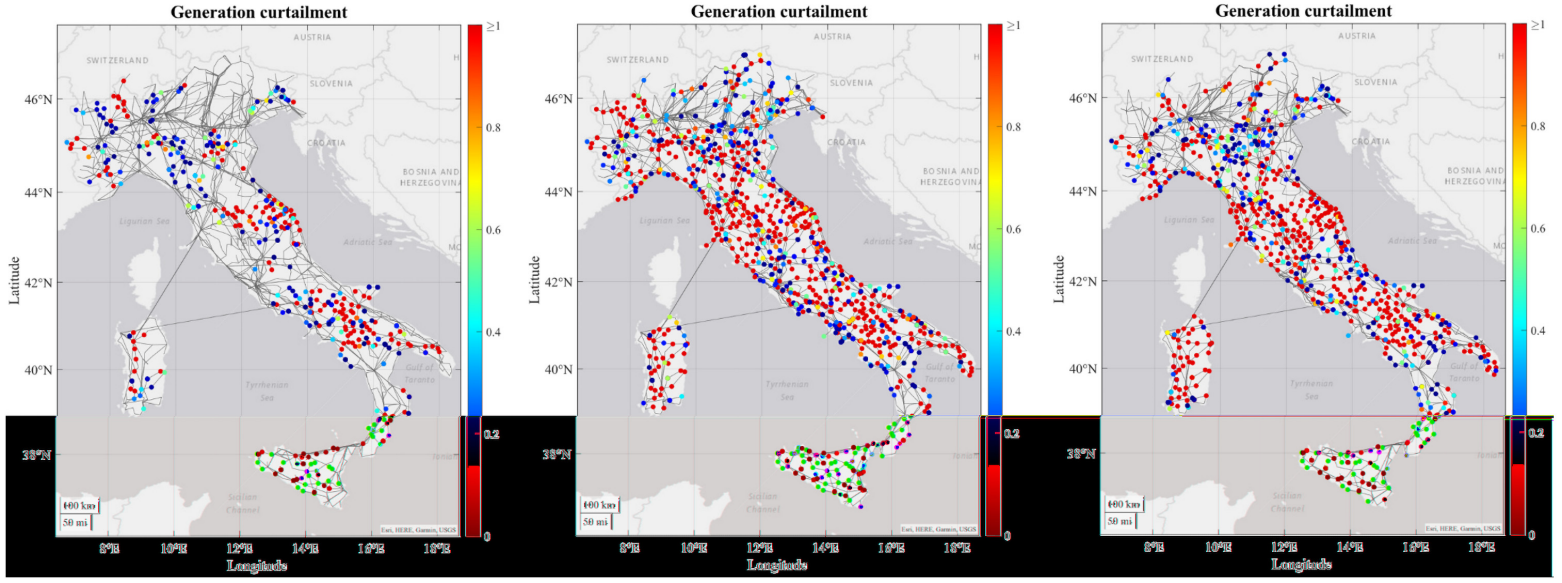
Development of generations congestions in the Italian regional case.

**Figure 4.**
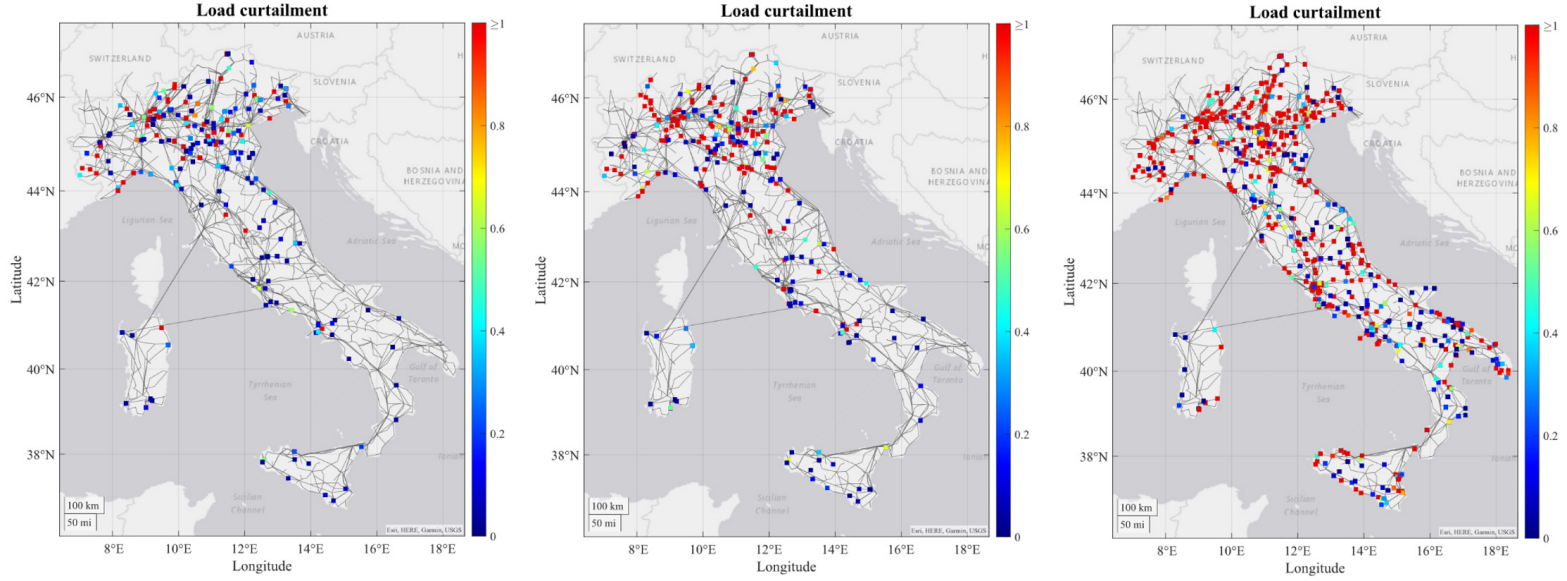
Development of load curtailment in the Italian regional case.

As regards to the simulation results, some tendencies can be observed for all or at least most of the RCs. For most of the RCs the number of congestions increase in each time horizon due to increasing load and generation profiles from 2030 to 2050, combining with the limitation on the maximum number of candidates that can be processed by the grid expansion tool, which means that some congestions may not be resolved and are transferred to the subsequent decades. In the figures below it can be seen how the severity of generation congestion ( Figure 3) and load curtailment ( Figure 4) in Italian RC change in each time horizon.

Concerning the number and the type of investment decisions, the number of candidates for traditional grid reinforcement (lines and transformers) in transmission networks is lower than in distribution networks, which means that more severe congestions occur in the distribution networks, and more than 42% of the transmission candidates are approved by the grid expansion tool. The only exception is the French region, which is a part of France and Benelux RC. The network for this RC was separated in order to decrease the simulation time. In this region, six traditional grid reinforcement candidates were manually added in the transmission network. They were not approved, nevertheless congestion had been eliminated by distribution candidates locally, along with storages candidates and load flexibilization in the congestion site. Regarding the storage and flexibility load candidates, overall, there is a trend to increase the percent of approval of the storage candidates from 2030 to 2040 and from 2040 to 2050, the average percent of approved candidates is 64%.

**Figure 5.**
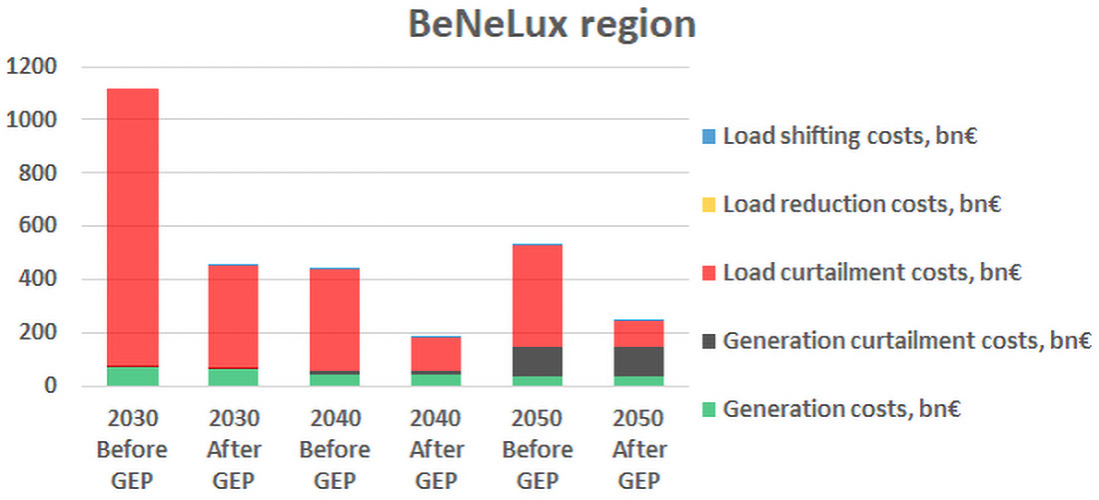
Results from the Benelux RC.

Additionally, the results for the change in the system dispatch costs before and after solving the grid expansion problem show that for most of the RCs the costs increase throughout the decades, which is mainly due to the limitation on the number of candidates that can be considered for each decade. However, for the Benelux RC, there is a significant decrease in the costs from 2030 to 2040, as shown in Figure 5, which is explained by the fact that the scenario of 2040 forecasts a significant increase in RES generation, whereas the load profile does not increase so drastically, and hence overall the load curtailment costs, which are the main contribution to the total costs in Benelux region, decrease in 2040 by approximately 68% comparing to 2030.

Also, for Northern Countries RC the total costs in 2040 decreased in comparison to 2030 because the approved candidates in 2030 significantly decreased the load curtailment by considerably relieving the congestion that would otherwise subsist in parts of the network.

With regard to the environmental impact assessment, it is clear that over the years the impact of carbon footprint plays a more significant role in the total costs across all RCs when compared with air quality costs.

## Results

### Learning from the RCs

***Identified opportunities for flexibility resources.*** The results of the six RCs show a great exploitation of flexibility resources in synergy with conventional expansion approach and presented in more details in FlexPlan report D5.2 ^23^. Indeed, every RC is characterized by the final selection of the different technologies (AC/DC branches, storage assets and flexible loads). For most of the RCs, the investments in storage and demand side management are frequently chosen as cost-effective solutions by the optimization algorithm, thus the potential of flexibility resources in the grid expansion planning is strongly supported by the simulation results.

In all three decades (2030, 2040 and 2050) and for all RCs, an overall reduction of the system costs is obtained when comparing costs coming from the OPF model (which carries out a pre-investment dispatching costs analysis) and the Grid Expansion Planning tool (which selects a subset of the proposed candidates able to minimize total system costs; this subset includes flexibility assets, working in synergy with conventional grid reinforcements).

The deployment of RES generation, which characterises the scenarios used as inputs for the FlexPlan tool, is shown to be very effective in cost reduction when the resources are geographically well-placed, i.e., installed in the regions where the consumption is high. Indeed, during weeks characterised by high local RES generation, load curtailment is reduced and, thanks to the strategic location (near the locations characterized by the highest consumption), the increase of energy production from RES does not produce additional congestions in the overall network.

***The importance of coordinated planning.*** The results of the simulations show of the importance of a coordinated planning of transmission and distribution networks. This can be seen mainly looking at three different aspects:

•  In many cases it is demonstrated that overall system costs which arise due to the presence of congestions in the transmission system are reduced thanks to the settlement of resources connected to the distribution network.

•  Often the acceptance of a candidate on a specific corridor also suggests the adjustment of close lines. Anyway, when lines at the border between transmission and distribution grids are considered, it is important to develop an integrated planning procedure in order to evaluate if the solution of a congestion on the transmission line determines the occurrence of a new congestion in the distribution network.

•  The selection of a candidate on the distribution or transmission network, could be beneficial for congestion which occurs respectively in the transmission or distribution network. In this case the cooperation during the development of the network expansion is necessary to avoid useless investments.

Finally, despite the high computational complexity of nodal models including T&D networks, some already mentioned features of the FlexPlan, especially the applied decomposition techniques, made it possible to retrain the numerical tractability of the models.

***Coordinated approach may create confidentiality-related issues.*** The above-mentioned coordinated approach of transmission and distribution planning processes proved to be very efficient, also in order to facilitate access and exchange of all necessary data. All parties performing a regulated task should be able to access data to an appropriate level of detail while respecting data privacy. For example, TSOs might need knowledge about consumption and generation at the point of common coupling between TSO and DSO, sometimes they also need information of the generation technology to understand if flexibility assets respect their obligations. Both long-term and operational planning should be conducted in coordination and, as long as confidentiality issues are met, even data concerning year ahead-availability plan, outages and emergency plans should be included.

***The authorisation procedures.*** The FlexPlan planning tool does not take into consideration authorization procedures and the time needed for an acceptance for the settlement of storage facilities and reinforcement/construction of transmission and distribution branches. In order to reinforce the network in the Nordic Region (only Norwegian Regulation is considered since accepted candidates are located in that country) planning candidates should only be built if they minimize the total socio-economic costs, which means that they are the most socio-economically profitable measure to meet the need and ensure compliance with laws and regulations among the evaluated possibilities.

Furthermore, possible negative impacts on the environment and on society are considered during the authorization procedure. The same goal is met by the FlexPlan planning approach, the target function is indeed based on the minimization of the overall system costs and environmental aspects are monetized directly in the target function.

***The energy storage.*** The importance of this issue was already highlighted during the screening phase, and thus has been carefully considered throughout the project. Indeed, Art. 36 and Art. 54 of IEM Directive 2019/944 ^5^ state that TSOs and DSOs are not allowed to own, develop, manage, or operate storage facilities and even if there are some exceptions concerning the ownership, in those conditions the installed capacity cannot be used for balancing and congestion services. Furthermore, some differences are found in the national implementations: in Italy, for instance, when no third parties are interested to develop the full request capacity, a TSO could be allowed to own the facility, but its operation must be assigned to third parties; the IEM Directive also foresees the TSO/DSO operation. The FlexPlan planning tool includes the integration of storage assets by means of a market procedure. As according to the identified regulatory limitations, storage facilities which are to be developed cannot be owned by system operators.

***Integration of flexible resources on a level playing field.*** As mentioned, flexibility assets are currently considered to be like traditional generation facilities. They are already allowed to participate according to all prequalification procedures developed. However, such procedures were mainly written considering technical characteristics of conventional power plants. Even if not explicitly, the allowance for the participation to electricity market without updating technical and operative parameters included in European and national regulations could create an unfavourable environment which hampers the integration of flexibility resources in the market. According to this, the FlexPlan consortium acknowledges that flexibility resources are not completely integrated on a level playing field and regulations of market participation should be updated to counteract the disadvantageous effects of the current regulatory framework. The reduction of the minimum power threshold for direct participation of demand-side resources to ancillary services markets as well as an adjustment of the products in these markets in order to comply with the characteristics of the flexible resources could be very beneficial. Another aspect to be considered is to provide a more stable, clear, and uniform regulation for aggregation throughout Europe and in thew single countries could help to ensure a viable business case for this important figure.

## Discussion: regulatory implications and the FlexPlan guidelines

Development of these recommendations is based on the importance of the role of flexibility resources, demonstrated by the FlexPlan tool, and possible regulatory barriers, identified throughout the project. In addition to several issues, which were pointed put already during the screening phase, several important learnings have been derived from the accomplished RCs.

### Tools for incentivisation of new flexibility resources

Following the recent regulatory developments, it is reasonable to assume that investments in storage and flexibility will remain mostly in the hands of investors, which are not associated with system operators. National regulatory authorities should translate the suitability of deploying new storage or flexibility in strategic network locations into opportune incentivization tools for potential investors. This complicates the traditional scheme, where system operators were the only subject entitled to invest after carrying out planning analyses.

Such incentivization tools should contain a locational element able to drive potential investors to prefer an investment in critical nodes, identified on the basis of the studies led by the system operators. This can be carried out either by means of locational capacity markets or by means of long-term contracts obliging the flexibility providers to reserve an adequate amount of capacity to be offered in services markets. However, the development of a long-term incentivising framework able to attract investments towards critical locations could reveal regions with high potential for the exercise of market power. In these cases, market-based mechanisms for the procurement of flexibility services should be combined with long-term contracts with a pre-established strike price, so as to discourage investors receiving long-term incentivisation to apply significant bid-up strategies. In alternative, a cap on bid prices could be explicitly established. Finally, a “must-run” situation, in which the system operator bids the asset on behalf of the owner can also be acceptable, but just in extreme cases.

Additionally, as already mentioned, real-time markets should be reformed by defining products that allow “flexibility” providers to compete with traditional resources on a “level playing field” basis. Operative constrains of storage and demand side management should be fully considered.

### Reforming the European electricity market

Following the previous topic, it is necessary to mention the market reforms being now investigated in Europe ^24^, so as to decouple market prices from gas prices (possibility of price-caps or two-stage markets) ^25^. These reforms, while considering the role of generators and loads, usually don’t consider explicitly the role of flexible resources (e.g., arbitrage between market prices at different times). Considering the fact that storage and demand side management will be two major players in the future provision of ancillary services, a clarification on the nature of the service provided by these subjects would bring to more forward-looking reform of market mechanisms.

### Regulatory framework for demand response and the role of aggregator

Despite some significant yet incremental steps done in the 2019/944 Directive, active use of demand response has been inhibited due to lack of a comprehensive regulatory framework for the subject. In that sense it is difficult to underestimate the importance of the forthcoming Network Code for Demand Response. The FlexPlan consortium acknowledges the significance of the framework guideline for the code presented by the European Union Agency for the Cooperation of Energy Regulators (ACER), which summarises the main subjects to be better detailed from the regulatory point of view. The final document shows a great improvement after the public consultation accomplished in autumn 2022. It also creates a logical connection between network development planning as described in Art. 32 ^5^ and demand response, as an alternative to system expansion.

Despite recognising the importance of aggregation for demand response, the 2019/944 Directive failed to define the role and responsibilities of the aggregator, the key element in the puzzle. Aggregation is a very demanding process because, not only it reduces the number of bids on the market, but also favours the integration of resources characterized by small capacities which would not be feasible to participate in the electricity market in other ways. The role and responsibilities of aggregators should be accurately designed within the redefinition of real-time market architectures. In the final version of the framework guideline more details have been specified, but the role of aggregator still remains somewhat unclear and probably has to be properly addressed on another legal level, e.g., in a new version of the 2019/944 Directive. Here, the FlexPlan consortium acknowledges that the possibility of an aggregator to compensate positions with opposite risk exposure can be considered as an element favouring their operation. However, the business case of the aggregators must also be considered so that their operation is capable to provide them with the needed revenues, without which no real subject, even in presence of a specific regulation, will ever volunteer to take such responsibility.

### Coordinated planning with a longer horizon

In future energy systems, TSO and DSOs should coordinate their planning activities. In fact, most of the potentially flexible loads as well as most distributed generation are being connected to distribution systems. However, it is not thinkable to allow a really integrated planning of transmission and distribution: on one side the optimization problem would be too complex and, on the other, system operators are not allowed to exchange private data with other subjects, be they even other system operators. Therefore, a coordinated approach can be suggested in which by means of an exchange of data at the border between different systems DSOs can, in case advantageous for the system, oversize their network so as to get fit to provide services to transmission. The T&D decomposition approach proposed by FlexPlan can be, in our opinion, a good starting point for reasoning on this approach.

In the future, planning studies should be carried out not for a single horizon year but over several decades in order to design the complete decarbonization pathway from mid to long-term (2030, 2040, 2050). To avoid getting sub-optimal results, privileging mid- over long-term goals or vice versa, the optimization should be carried out in a coordinated way between the different decades, as proposed by the FlexPlan project.

### Updates for cost-benefit analyses and internalisation of environmental costs

CBAs must consider positive effects of flexibility resources. The uptake of flexibility resources requires an update of the present CBA approach which should consider every benefit brought by flexibility resources. Two main aspects should be considered while performing a CBA: first the coordination between TSO and DSOs in defining required investment for the network reliability and secondly the monetization of all factors should be strained. In the FlexPlan approach, environmental aspects and carbon-footprint are monetized and T&D decomposition is developed in order to allow a coordinated CBA between different System Operators. Key importance must be attributed to greenhouse gases and other pollutant reduction. Environmental aspects should be put in monetary terms so that they can be co-evaluated with more traditional ones (social welfare, etc).

## Conclusions

This article identifies the opportunities coming from the deployment of flexibility resources during the expansion planning process and evaluates the impact of the regulatory framework in terms of possible barriers and limitations. Based on the six RCs analysed by FlexPlan, the study proposes a set of regulatory guidelines to improve use of flexible resources during expansion planning procedures and thereby to achieve more efficient operation of the distribution and transmission networks with high share of renewable generation.

## Data Availability

The simulation data cannot be made public, since input data arrive from the ENTSO-E TYNDP model for which the Consortium signed a non-disclosure agreement. The reader should apply for individual access to the data directly with ENTSO-E.

## Software availability

The FlexPlan toolbox is not public but the Flexplan.jl library replicates all modelling functionalities of the grid expansion planning tool, but not those of the pre-processor. Installation instructions, information regarding problem types and network formulations are provided in the package documentation ( https://electa-git.github.io/FlexPlan.jl/dev/).

**Source code for the optimal system routing library available from:** https://github.com/Electa-Git/OptimalTransmissionRouting.jl

**Source code for the open access license toolbox available from:** https://github.com/Electa-Git/FlexPlan.jl

**Archived source code at time of publication:** https://doi.org/10.5281/zenodo.8035492 ^26^

**License:** BSD 3-Clause License
