# Supplementary material for: Regulatory Implications for considering flexible resources in network expansion planning: main lessons from regional cases in the H2020 FlexPlan project
Source: Open Res Eur. 2024 Apr 29;3:149. Originally published 2023 Sep 15. [Version 3] doi: 10.12688/openreseurope.15873.3 (PMC10966340; doi:10.12688/openreseurope.15873.3)
Supplement: Peer review history

# Reviewer report: Reviewer response for version 2

**DOI:** 10.21956/openreseurope.18477.r37570

**Published:** 2024-03-26

**Authors:** Ali Moradi Amani

**Affiliations:** 1 RMIT University, Melbourne, Victoria, Australia

This article explores the potential benefits arising from integrating flexibility resources, e.g. demand response programs and storage systems, into the expansion planning phase of power grids, while also assessing how regulatory frameworks might pose barriers or limitations. Based on the analysis conducted by the FlexPlan project in six Regional Cases (RCs), the study offers regulatory recommendations aimed at enhancing the utilization of flexible resources in expansion planning processes and coordination of transmission and distribution planning. Ultimately, the goal is to foster more efficient operation of distribution and transmission networks, particularly in contexts with significant renewable energy generation.

 The paper is generally well-structured and well-written, and addresses a very timely problem of integrating flexible resources to make the whole grid reliable. The figures are also informative. I have three minor suggestions for authors:

1. Although the optimisation problem has been explained narratively, it requires more expansion. I suggest bringing more detail on the objective function and constraints, even in a simple mathematical form if it helps.
2. As the proposed methodology has been applied to joint transmission and distribution grid, readers may be interested in the scalability of the optimisation problem and how it can deal with large-scale systems. Please elaborate more on it.
3. I guess electric vehicles have not been in the scope of FlexPlan as they have not been addressed as flexible resources. EV charging, through smart algorithms, need also relevant regulatory framework and should be considered. Authors can at least acknowledge EVs in the conclusion for future works.

Is the study design appropriate and does the work have academic merit?

Yes

Is the work clearly and accurately presented and does it cite the current literature?

Yes

If applicable, is the statistical analysis and its interpretation appropriate?

Not applicable

Are all the source data underlying the results available to ensure full reproducibility?

Partly

Are the conclusions drawn adequately supported by the results?

Yes

Are sufficient details of methods and analysis provided to allow replication by others?

Yes

Reviewer Expertise:

Control systems, DER-dominant power grids

I confirm that I have read this submission and believe that I have an appropriate level of expertise to confirm that it is of an acceptable scientific standard.

# Reviewer report: Reviewer response for version 2

**DOI:** 10.21956/openreseurope.18477.r36906

**Published:** 2024-02-26

**Authors:** Sina Ghaemi

**Affiliations:** 1 Aalborg University, Aalborg, Denmark

Authors in this paper, within the FlexPlan Horizon 2020 project, have addressed the regulatory implications of considering flexible assets such as storage and flexible loads in the optimal expansion planning of transmission and distribution networks for three different horizons: namely 2030, 2040, and 2050. The paper has been prepared with a well-structured approach, and the authors' way of addressing this issue seems comprehensive and precise. They have started by reviewing the Pan-European regulatory framework and expressing how this project aligns with the Pan-European political objectives. Then, the introduction of the model for optimal planning of both transmission and distribution grids has been presented. Finally, based on the findings of their simulation models, some recommendations for the existing Pan-European level regulation have been provided. The topic is timely and relevant to the existing challenges in the energy transition. However, the reviewer’s comments can be sorted as follows:

 1. According to the methodology introduced in this paper, the FlexPlan pre-processor specifies the list of candidates for investment, which is based on computing locational marginal price (LMP). The authors need to explain this part a bit more. Have the authors considered computing LMP at both transmission and distribution grids to detect potential places for upgrades? In addition, have the authors considered a priority list in their potential candidates based on the reduction in the absolute value of LMP and its frequency of being high in terms of absolute value?

 2. The authors also need to briefly describe what mechanisms have been applied for empowering demand flexibility. Have the authors considered incentives for flexible loads' contribution in providing flexibility? In the case of considering incentives, are they consistent for each year within each planning horizon?

 3. The minor comment of the reviewer is related to Figure 5 of the paper, which can be replaced with one of higher quality.

Is the study design appropriate and does the work have academic merit?

Yes

Is the work clearly and accurately presented and does it cite the current literature?

Yes

If applicable, is the statistical analysis and its interpretation appropriate?

I cannot comment. A qualified statistician is required.

Are all the source data underlying the results available to ensure full reproducibility?

Yes

Are the conclusions drawn adequately supported by the results?

Yes

Are sufficient details of methods and analysis provided to allow replication by others?

Yes

Reviewer Expertise:

Integrated energy systems, Hydrogen economy, energy markets and Renewable energy

I confirm that I have read this submission and believe that I have an appropriate level of expertise to confirm that it is of an acceptable scientific standard, however I have significant reservations, as outlined above.

# Response

**Published:** 2024-03-31

**Authors:** Andrei MORCH

**Affiliations:** SINTEF Energy Research, Norway

1. The present paper focuses on the regulatory implications for considering the flexible resources in the network planning process. Very detailed explanation of the pre-processor and its integration with the FlexPlan planning tool has been previously presented in several publications, where https://doi.org/10.5281/zenodo.7064973 (COGRÉ 2022) is the most accessible. In the 3rd version an additional sentence has been added to the section “FlexPlan Methodology” and the CIGRÉ 2022 paper has been added to the list of references.
2. The FlexPlan project did not include physical demonstrations sites, so the testing and refinement of the FlexPlan planning tool was done via six regional case studies, as it is described in the article. The necessity for and ways for improving incentivisation of new flexible resources is described in Section “Tools for incentivisation of new flexibility resources”.
3. An enhanced version of Figure 5 has been uploaded.

# Reviewer report: Reviewer response for version 2

**DOI:** 10.21956/openreseurope.18477.r37312

**Published:** 2024-01-29

**Authors:** Hossein Farahmand

**Affiliations:** 1 Norwegian University of Science and Technology, Trondheim, Norway

I acknowledge the authors' efforts to improve the article. They have effectively addressed all of my comments, and I am confident that the paper can be concluded in its current state.

Is the study design appropriate and does the work have academic merit?

Yes

Is the work clearly and accurately presented and does it cite the current literature?

Yes

If applicable, is the statistical analysis and its interpretation appropriate?

I cannot comment. A qualified statistician is required.

Are all the source data underlying the results available to ensure full reproducibility?

Yes

Are the conclusions drawn adequately supported by the results?

Yes

Are sufficient details of methods and analysis provided to allow replication by others?

Yes

Reviewer Expertise:

Local flexibility markets and flexibility operation in smart systems; Power System balancing; Power system operation and planning; Power Market

I confirm that I have read this submission and believe that I have an appropriate level of expertise to confirm that it is of an acceptable scientific standard.

# Reviewer report: Reviewer response for version 1

**DOI:** 10.21956/openreseurope.17147.r35159

**Published:** 2023-10-19

**Authors:** Hossein Farahmand

**Affiliations:** 1 Norwegian University of Science and Technology, Trondheim, Norway

This paper focuses on the regulatory challenges encountered during FlexPlan's initiation phase and how these were refined by examining regional cases in the European power system. The paper provides a concise overview of the FlexPlan methodology and its practical application in these regional contexts. The FlexPlan Horizon2020 project aimed to reinforce grid planning by incorporating novel storage and flexibility resource considerations for electricity transmission and distribution grids. This approach aimed to provide an alternative to constructing new grid components, aligning with the European Commission's "Clean Energy for all Europeans" regulatory package. To ensure the applicability of these innovations, FlexPlan developed six regional case studies spanning across Europe. These cases provided valuable insights into grid planning considerations up to the year 2050. Overall, the manuscript is well written. The technical content of the paper is practical and has academic merit. The subsequent suggestions can be taken into account to further improve the quality of this work:

- I suggest to include a discussion on the impact of the uncertain generation of VRES and the subsequent load variation patterns on transmission expansion planning decisions. I believe it is essential to discuss the robustness of the expansion choices. Robustness can pertain to the capacity of the grid expansion plan to effectively accommodate a range of uncertainties and evolving conditions over an extended period.
- Please specify the corresponding year for the generation curtailment and load curtailment data presented in Figures 3 and 4 for Italy.
- Avoid informal language, i.e., “However, it is not thinkable to allow a really integrated planning of transmission and distribution.”
- Please elaborate on this sentence: “Here, the FlexPlan consortium acknowledges that the possibility of an aggregator to compensate positions with opposite risk exposure can be considered an element favouring their operation”.
- Briefly explain how the FlexPlan project monetizes environmental factors and assesses carbon footprints.

Is the study design appropriate and does the work have academic merit?

Yes

Is the work clearly and accurately presented and does it cite the current literature?

Yes

If applicable, is the statistical analysis and its interpretation appropriate?

I cannot comment. A qualified statistician is required.

Are all the source data underlying the results available to ensure full reproducibility?

Yes

Are the conclusions drawn adequately supported by the results?

Yes

Are sufficient details of methods and analysis provided to allow replication by others?

Yes

Reviewer Expertise:

Local flexibility markets and flexibility operation in smart systems; Power System balancing; Power system operation and planning; Power Market

I confirm that I have read this submission and believe that I have an appropriate level of expertise to confirm that it is of an acceptable scientific standard, however I have significant reservations, as outlined above.

# Response

**Published:** 2024-01-02

**Authors:** Andrei MORCH

**Affiliations:** SINTEF Energy Research, Norway

Thank you for your prompt and constructive review. The comments have been addressed as follows:

1. Explanation added to the text: (in brief) due to complexity of the models and the lack of computational resources, it was considered to simulate only one scenario (one paragraph added).
2. Added simulation years 2030/2040/2050 to the captions for Figure 3 and 4.
3. “Thinkable” replaced with “conceivable”.
4. The sentence has been re-written.
5. Explanation related to monetisation of the environmental costs and corresponding reference and corresponding reference to previous research (doi: 10.1109/PTC.2015.7232638)
